# Coexisting Meningioma and Glioma in the Same Patient: A Case Report

**DOI:** 10.7759/cureus.64543

**Published:** 2024-07-14

**Authors:** Rey Alexis Sususco, Marietta Olaivar

**Affiliations:** 1 Section of Adult Neurology, Cardinal Santos Medical Center, San Juan, PHL; 2 Department of Neuroscience, University of the East Ramon Magsaysay Memorial Medical Center, Quezon City, PHL

**Keywords:** intracranial neoplasm, neoplasm, malignant, benign, glioblastoma, meningioma, collision tumors

## Abstract

The simultaneous occurrence of two distinct intracranial neoplasms within a single patient presents a unique and exceedingly rare clinical scenario. Such cases pose significant diagnostic and therapeutic challenges, complicating clinical decision-making and treatment planning. Understanding the intricacies of these cases is crucial for improving patient outcomes and advancing clinical knowledge. This report aims to provide valuable insights into the complexities surrounding the management of patients with coexisting different intracranial neoplasms. This case report seeks to enhance the understanding of healthcare professionals dealing with similar cases and contribute to the existing literature on this rare phenomenon. This case report details a patient harboring two different but coexisting intracranial neoplasms. The patient, a 73-year-old woman, presented with behavioral changes and bilateral leg weakness. Initial imaging studies, including CT and MRI scans, revealed the presence of two distinct intracranial masses located in the frontal lobes and the falx cerebri. Neurosurgical, chemotherapy, and radiotherapy were offered; however, the patient’s family opted for palliative care. The concurrent presence of two distinct intracranial neoplasms in a single patient underscores the complexity of such cases. This report highlights the diagnostic and therapeutic challenges encountered and the importance of a multidisciplinary approach to managing these patients. By sharing this case, we hope to contribute to a broader understanding of such rare clinical scenarios and aid in developing more effective management strategies in the future.

## Introduction

Intracranial neoplasms encompass a wide range of benign and malignant tumors originating within various intracranial structures, including the brain parenchyma, meninges, cranial nerves, and pituitary gland, thus presenting with diverse clinical manifestations that may mimic other neurological conditions. The occurrence of multiple intracranial neoplasms within a single patient is an exceptionally rare phenomenon and poses a clinical challenge, necessitating a meticulous diagnostic and therapeutic approach encompassing neuroimaging, histopathological examination, and molecular profiling [1].

We present the case of a 73-year-old woman who exhibited symptoms suggestive of intracranial pathology, with radiological imaging showing two distinct brain tumors. We aim to contribute to the limited knowledge surrounding this uncommon condition, emphasizing the need for personalized, multidisciplinary care to optimize patient outcomes.

## Case presentation

**Figure 1:**
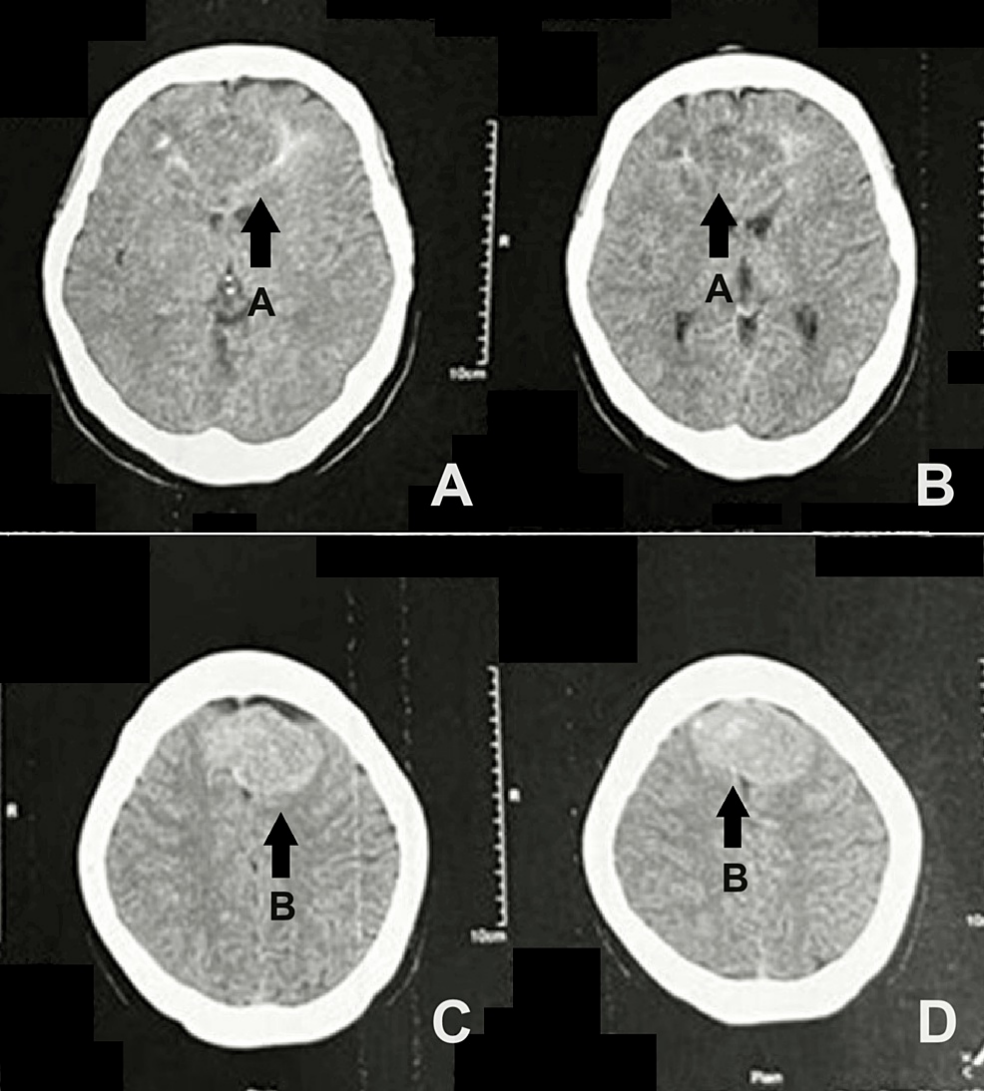
Axial cranial CT scan without contrast of the patient. Arrow A in panels A and B points to a fairly defined, heterogeneous, intra-axial mass measuring 3.1x4.2x3.4 cm, crossing the midline in both frontal lobes with perilesional edema. Arrow B in panels C and D points to a well-defined, iso- to slightly hyperdense, extra-axial mass adjacent to the falx cerebri measuring 3.4x4.8x3.4 cm, with minimal calcifications in the mid-frontal convexity with minimal perilesional edema.

A 73-year-old female presented to the ER with a five-month history of progressive behavioral changes, initially characterized by irritability, decreased social interaction, and mild memory lapses. The symptoms were initially attributed to aging. Over time, weakness and worsening memory lapses developed, leading to consultation with consideration of stroke without further investigation. Ten days prior to admission, the patient experienced urinary and fecal incontinence, prompting a neurology consultation and a cranial CT scan revealing a fairly defined, heterogeneous, intra-axial mass measuring 3.1x4.2x3.4 cm crossing the midline in both frontal lobes with perilesional edema and a well-defined, iso- to slightly hyperdense, extra-axial mass adjacent to the falx cerebri measuring 3.4x4.8x3.4 cm, with minimal calcifications in the mid-frontal convexity with minimal perilesional edema (Figure 1). Immediate hospital admission was recommended but declined by the family. Twelve hours prior to admission, the symptoms worsened with decreased verbal output, prompting an ER consultation. The patient had a history of hypertension and a family history of hypertension and cardiovascular diseases. No history of smoking, drinking, or illicit drug use was reported.

Upon examination, significant findings include stable vitals, the patient being lethargic but was arousable to tapping and able to sustain eye-opening. She displayed regard and was minimally conversant. However, her responses were slow, and she demonstrated signs of bradyphrenia, often nodding or shaking her head when answering questions. She exhibited a dysthymic mood with a blunted affect and showed poor attention, concentration, insight, and judgment. She was oriented to self and place but not to time. Her recent and remote memory remained intact but had impaired immediate and delayed memory recall. There was no evidence of Gertsmann syndrome (acalculia, agraphia, finger agnosia, and left-right confusion). Testing for calculation, drawing, stereognosis, and graphesthesia was done, but accurate assessment was hindered by slow replies and generalized weakness. There were no cranial nerve abnormalities. The motor examination revealed weakness with a grading of 4/5 over the upper extremities and noticeably weaker lower extremities with a grading of 2/5 on the right and 3/5 on the left. The patient denied any sensory deficit. The muscle stretch reflexes were unremarkable, but there were positive palmomental and Babinski reflexes on the right. The cerebellar and meningeal examinations were unremarkable.

**Figure 2:**
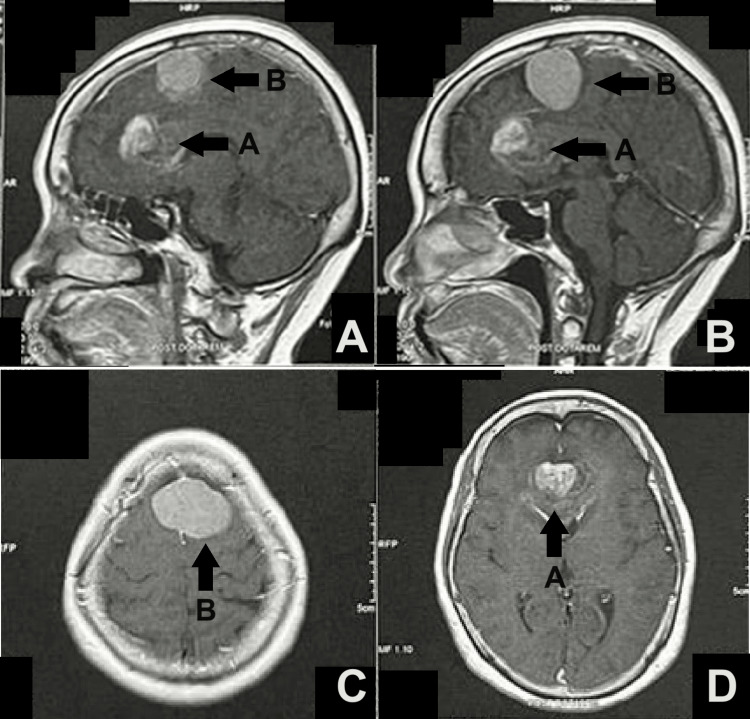
Cranial MRI of the patient, with and without contrast. The sagittal view in panels A and B shows arrow A pointing to an irregularly shaped butterfly tumor involving the callosal genu and the cingulate gyri, measuring 3.2x4.3x3.2 cm with a moderate amount of infiltrative edema. The axial view in panels C and D shows arrow B pointing to a separate homogeneously enhancing extra-axial mass, noted to straddle the high frontal interhemispheric falx, measuring 3.4x4.6x3.5 cm, appearing to infiltrate the middle third of the superior sagittal sinus and demonstrated no significant vasogenic edema.

A cranial MRI was performed (Figure 2), which revealed an irregularly shaped butterfly tumor, most likely a glioblastoma (GBM), involving the callosal genu and the cingulate gyri. It measured 3.2x4.3x3.2 cm in the anteroposterior (AP), transverse, and craniocaudal dimensions and with a moderate amount of infiltrative edema. A separate, homogeneously enhancing extra-axial mass, most likely a meningioma, was noted to straddle the high frontal interhemispheric falx, measuring 3.4x4.6x3.5 cm, appearing to infiltrate the middle third of the superior sagittal sinus and demonstrated no significant vasogenic edema. Further laboratory tests were done (Table 1), which were all unremarkable.

**Table 1:** Clinical laboratory results of the patient. eGFR, estimated glomerular filtration rate; SGOT, serum glutamic-oxaloacetic transaminase; SGPT, serum glutamate pyruvate transaminase; WBC, white blood cells; INR, international normalized ratio; PTT, partial thromboplastin time; RBC, red blood cells.

Tests	Results	Reference Range
Sodium	132	135-148 mmol/L
Potassium	4.1	3.5-5.3 mmol/L
Magnesium	0.99	0.63-1.05 mmol/L
Ionized calcium	1.09	1.13-1.31 mmol/L
Blood urea nitrogen	3.69	2.1-7.1 mmol/L
Creatinine	56.76	49-90 µmol/L
eGFR	89	>60 mL/min/1.73m^2^
SGOT	15.73	<40 IU/L
SGPT	17.6	<40 IU/L
Hematology		
Hemoglobin	129	120-140 g/L
Hematocrit	38	37-47%
WBC	8.8	5-10x10^9^/L
Neutrophils	66	37-72%
Lymphocytes	32	20-50%
Monocytes	1	0-14%
Eosinophils	1	0-6%
Platelet	269	150-440x10^12^/L
Prothrombin time	11.4	10-13 seconds
Control	12	-
INR	0.95	-
Activity	96%	-
PTT	23.6	29-34 seconds
Control	30	-
Urinalysis		
Color	Light yellow	Varying degrees of yellow
Turbidity	Slightly hazy	Clear
Leukocytes	Negative	Negative
Nitrite	Negative	Negative
Protein	Trace	Negative
Blood	Positive	Negative
Specific gravity	1.01	1.005-1.030
Ketone	Negative	Negative
Glucose	Negative	Negative
RBC	9-10	0-1
WBC	1-2	0-5
Bacteria	Rare	-
Epithelial cells	Few	-
Mucus threads	Few	-

The patient was admitted to the neurology department and referred to neurosurgery. Dexamethasone (5 mg IV every six hours) was initiated to alleviate vasogenic edema, while maintenance medication of losartan (50 mg/tab once daily) was continued. Plans were made for bifrontal craniotomy and mass excision for histopathological examination, with a preoperative assessment conducted by the IM-cardiology service, determining an intermediate risk for intraoperative complications. Over the course of the hospital stay, the patient’s condition stabilized, and the patient showed neurological improvement (improved attention and concentration, negative Babinski sign, and no recurrence of incontinence) and improved motor strength. Despite stable vital signs, the family declined surgery, leading to referral to radiation oncology and palliative medicine. Plans for discharge and outpatient palliative radiation therapy were arranged. On the ninth day of hospitalization, the patient was discharged with the following medications: dexamethasone 4 mg/tab, one tablet thrice a day (tapering over one month), losartan 50 mg/tab daily, lactulose 30 mL daily, calcium tablet daily, and omeprazole 40 mg/tab daily. A close outpatient follow-up was advised.

## Discussion

Gliomas are most commonly diagnosed in middle-aged adults, with the average age of onset for GBM being approximately 60 years. However, gliomas can affect individuals of any age. Men have a greater incidence rate. Most high-grade gliomas occur randomly, without a genetic predisposition. Many of these tumors originate in the deep white matter as a diverse mass and rapidly spread across the brain, occasionally reaching significant dimensions before receiving medical attention. Conversely, meningiomas account for approximately one-third of all primary tumors in the brain. They are more prevalent in women than in men and are most diagnosed in individuals between their 60s and 70s. Some have a familial nature [2].

In a case report by Suzuki et al. (2010), concurrent glioma and meningioma have been reported, with some suggesting it could be a coincidence rather than a shared pathway abnormality [3]. However, adjacent double tumors hint at a potential common causal background or condition. The theory proposes that if the presence of neighboring double tumors is due to a common underlying cause, signal transduction pathways may play a crucial role in the development of these tumors. The varying rates at which the two tumors grow indicate that meningioma may occur before GBM, causing it by impairing the p53 protein and activating receptor tyrosine kinases (RTKs). Pathway anomalies may also contribute to the development of neuronal and oligodendroglial cells in GBM tissues [4].

Temporal GBMs comprise 24.8% of all GBMs, while sphenoid ridge and temporal convexity meningiomas account for 13.2% of all meningiomas, according to the Brain Tumor Registry of Japan. The occurrence of simultaneous temporal GBM and meningioma at the same location is estimated to be approximately 0.32 per 10 billion individuals annually, translating to roughly one person per five years among the worldwide population of 6.8 billion [5]. Overall, the prevalence of GBM and meningioma in neighboring locations is uncommon. The immunohistochemical examination demonstrated distinct differentiation in GBMs and activation of signaling pathways in both tumors, indicating a potential malfunction in p53 that promotes tumor advancement. Paracrine signaling mediated by platelet-derived growth factor (PDGF) may contribute to the induction of one tumor from another [6].

Another case report by Al Mashani et al. (2017) reiterates the rarity of a coexisting meningioma and glioma in a single patient. Multiple neoplasms of different pathologies are rare entities, with some cases showing a meningioma as the initial lesion that later develops into a glioma over time. Patients can present with multiple symptoms, such as headache, dysarthria, memory disturbances, and hemiparesis, as per the location of the lesion [7].

Accurate preoperative diagnosis can be challenging due to their distinct histopathological features and overlapping radiological characteristics. Meningiomas are typically well-circumscribed, extra-axial tumors originating from the arachnoid cells, while GBM is a highly infiltrative, intra-axial tumor of glial origin. Advanced neuroimaging techniques, including MRI with contrast enhancement and spectroscopy, are crucial for characterizing the different components of these tumors. Histopathological evaluation remains the gold standard for definitive diagnosis, highlighting the importance of obtaining samples from both tumor entities. However, this was not performed in the patient’s case [8]. Nestler et al. (2007) emphasized that genetic testing of tumor cells should be performed when different histological types are present in a close spatial relationship. Understanding the distinct molecular signatures of each tumor type may have implications for targeted therapies and provide prognostic information [9].

The best treatment for coexisting meningioma and GBM is not well-established and requires a tailored approach. Surgical resection remains fundamental, aiming to remove both tumors safely. Some prefer separate surgeries if the tumor locations are relatively far from each other, while others monitor closely and operate only if the tumors cause symptoms. Some perform simultaneous surgery if the tumors are close. Adjuvant therapies, including radiotherapy and chemotherapy with agents such as temozolomide, are crucial for survival, particularly for GBM. However, the synergistic or antagonistic effects on coexisting tumors warrant further investigation [10].

The prognosis for patients with both meningioma and GBM depends on tumor grade, extent, patient age, health, and surgical success. The survival rates vary widely but are generally worse than for either tumor alone, and the presence of coexisting tumors can have an impact on the management and outcomes of both tumors [11].

## Conclusions

The simultaneous occurrence of a meningioma and a GBM multiforme is a rare and challenging clinical scenario. An illustration of the intricate interaction that takes place between the several neoplastic entities that are located within the intracranial compartment is provided by this situation. For its assessment and management, it is necessary to conduct a comprehensive diagnostic evaluation and choose a strategy that considers several different fields of study.

The example presented can have a substantial influence on future studies since it sheds insight into the possible complexities and connections between different types of intracranial neoplasms. By providing thorough clinical observations, diagnostic challenges, and therapeutic possibilities, this work can stimulate further research into the underlying genetic, molecular, and environmental factors causing such co-occurrences. Additionally, it might pave the way for more in-depth studies on customized treatment regimens and long-term outcomes, ultimately improving patient outcomes and advancing neuro-oncology.
